# Geometrical Changes of the Aorta as Predictors for Thromboembolic Events After EVAR With the Anaconda Stent-Graft

**DOI:** 10.1177/15266028221105839

**Published:** 2022-07-02

**Authors:** Jaimy A. Simmering, Mattijs de Vries, Marieke Haalboom, Michel M. P. J. Reijnen, Cornelis H. Slump, Robert H. Geelkerken

**Affiliations:** 1 Division of Vascular Surgery, Department of Surgery, Medisch Spectrum Twente, Enschede, The Netherlands; 2 Multi-Modality Medical Imaging (M3i) Group, Faculty of Science and Technology, Technical Medical Centre, University of Twente, Enschede, The Netherlands; 3 Division of Vascular Surgery, Department of Surgery, Diakonessenhuis Utrecht, Utrecht, The Netherlands; 4 Medical School Twente, Medisch Spectrum Twente, Enschede, The Netherlands; 5 Division of Vascular Surgery, Department of Surgery, Rijnstate Hospital, Arnhem, The Netherlands; 6 Robotics and Mechatronics (RaM) Group, Faculty of Electrical Engineering, Mathematics and Computer Science, Technical Medical Centre, University of Twente, Enschede, The Netherlands

**Keywords:** Anaconda stent-graft, geometrical parameters, EVAR, computed tomography, limb graft occlusion, thromboembolic event

## Abstract

**Purpose:**

Thromboembolic events (TE), including limb graft occlusion (LGO) and distal limb embolization (DLE), are common complications after endovascular aneurysm repair (EVAR). The aim of this study was to find predictors for TE in patients treated with the Anaconda stent-graft for infrarenal aneurysms.

**Materials and Methods:**

Geometrical and anatomical variables were retrospectively analyzed in a consecutive Anaconda cohort. Pre- and postoperative CT scans were used to derive geometrical parameters length, curvature, torsion, and tortuosity index (TI) from the center lumen lines (CLLs). Limb characteristics, pre-to-post EVAR and mid-term-follow-up changes in the parameters were evaluated for their predictive value for TE.

**Results:**

Eighty-four patients (mean age 74±8.3 years, 74 men) were enrolled. The risk of TE was lowered with pre-to-post implant decreasing TI (steps of 0.05: OR: 1.30, 95% CI: 1.01-1.66, p=0.04), pre-to-post implant decreasing mean curvature (OR: 1.08, 95% CI: 1.01-1.16, p=0.03), and a larger degree of circumferential common iliac artery (CIA) calcification (OR: 0.98, 95% CI: 0.97-1.00, p=0.03). The only LGO predictor was the caudal relocation of maximal curvature after EVAR (OR: 1.01, 95% CI: 1.00-1.01, p=0.04). Preventors of DLE were CIA diameter (OR: 0.87, 95% CI: 0.76-0.99, p=0.04), circumferential CIA calcification (OR: 0.97, 95% CI: 0.95-1.00, p=0.03), mean and maximal curvature of the preoperative aortoiliac trajectory (OR: 0.86, 95% CI: 0.79-0.94, p<0.01 and OR: 0.97, 95% CI: 0.95-1.00, p=0.03, respectively) and pre-to-postoperative decrease in mean curvature (OR: 1.11, 95% CI: 1.02-1.21, p=0.02). Midterm TE predictors were length (OR: 0.95, 95% CI: 0.89-1.01, p=0.08) and torsion maximum location (OR: 1.01, 95% CI: 0.99-1.01, p=0.10).

**Conclusion:**

The present study confirms that treatment of infrarenal AAA with an Anaconda stent-graft is related to a relatively high TE rate which decreases with a pre-to-postoperative reduction in curvature and TI, and a larger degree of circumferential CIA calcification. In other words, more aortoiliac straightening and more circumferential CIA calcification may prevent TE development after EVAR with this stent-graft.

## Introduction

The Anaconda stent-graft (Terumo Aortic, Inchinnan, Scotland, UK) performs in line with other stent-grafts for infrarenal aneurysms on sealing, migration, endoleaks, and treatment success even in more challenging anatomy with neck angulation up to 90° whereas comparable devices such as Endurant (Medtronic Vascular, Inc, Minneapolis, MN, USA), Excluder (W. L. Gore & Associates, Flagstaff, AZ, USA), Zenith (Cook Medical Inc., Bloomington, IN, USA), and TREO (Terumo Aortic, Sunrise, FL, USA), are indicated up to 75° angulation.^1–5^ On the other hand, the reported limb occlusion rate after endovascular aneurysm repair (EVAR) with Anaconda seems to be high.^1–3,5–8^ The limb occlusion rate after 5 to 6 years is 3.5% to 9.8%^1,2,5,9,10^ in Anaconda stent-grafts, compared to 3.3% to 5.3%,^1,3,8,11^ 2.2% to 12.4%,^1,11–13^ and 0.7% to 3.3%^1,3,11^ for respectively Endurant, Zenith, and Excluder stent-grafts. Suggested technical predisposing factors for limb occlusion are the omission of intraoperative ballooning of the stent-graft and extension into the external iliac artery.^5,11^ Known predisposing aortoiliac geometry for limb occlusion after EVAR are strong angulation, severe iliac calcification, and small distal limb diameter.^5,14–16^ Nevertheless, the pathophysiology behind the elevated limb occlusion rate in Anaconda stent-grafts is not yet understood. In the present study, we analyze geometrical variables in a consecutive single center Anaconda stent-graft cohort with the aim to determine whether geometrical variables can be identified as risk factors for limb occlusion.

## Materials and Methods

### Study Design

In this single center retrospective study, all consecutive abdominal aortic aneurysm (AAA) patients treated with an off-the-shelf Anaconda stent-graft from December 2014 until December 2018 were included. Patients treated with a single limb or aorto-uni-iliac Anaconda or treated for non-aneurysmal disease of the aortoiliac trajectory were excluded. Patient information, including demographics, comorbidities, and outcome variables were obtained until June 2020 and reported according to the reporting standards for EVAR.^17^

The study protocol was approved by the institutional review board (K18-43) and the regulations from the Dutch Act on Medical Scientific Research Involving Human Beings (WMO) and General Data Protection Regulation (AVG) were followed when using the medical data. Consent for research participation was waived as it was a retrospective study.

Pre- and postoperative computed tomography (CT) scans were collected for detailed analysis of geometrical changes. The local follow-up protocol included 1 postoperative CT within 3 months after EVAR and thereafter follow-up by ultrasound. Additional CT scans were also included, regardless the objective of the CT scan, as long as the abdominal aortoiliac trajectory was imaged. When more than 1 preoperative CT scan was available the scan acquired closest to the date of the primary intervention was used for analysis; when more than 2 postoperative CT scans were available the first postoperative scan *and* the scan closest to 1 year after EVAR were used (midterm follow-up).

The included patients were divided into subgroups. Patients diagnosed with limb occlusion of the device, defined as total device limb occlusion and/or symptomatic stenosis, anytime during the follow-up period were categorized in the limb graft occlusion (LGO) group. Patients that presented with a symptomatic and imaging confirmed peripheral embolism diagnosed to originate from the stent-graft after excluding other potential sources, such as cardiac or thoracic aortic pathology, were included in the distal limb embolization (DLE) group. The LGO and DLE groups were combined in the thromboembolic event (TE) group. Patients without any TE were categorized in the patent-group (PG).

The primary objective of this study was to observe the differences between the PG and TE groups in limb characteristics and the change in geometrical parameters from the preoperative to first postoperative CT scans. Moreover, potential predictive values of these metrics for TE were determined. Midterm geometrical changes in the geometrical variables were compared between groups as the difference from first postoperative CT to the 1-year follow-up CT. Secondary endpoints were to compare PG vs. LGO and PG vs. DLE and evaluate potential predictive variables for LGO and DLE, respectively.

### Image Acquisition

The CT scans were performed on a Somatom Definition AS scanner (Siemens, Healthcare, Erlangen, Germany). The scan parameters included rotation time 0.5 seconds, tube potentials 120 kV, tube current 60 to 65 mAs, collimation 64 mm x 0.6 mm, pitch factor 0.9 to 1.4, reconstruction matrix size 512 x 512 pixels and slice thickness ranged between 1.0 and 3.0 mm, obtained using reconstruction kernel I31f, I25f, or I41f Medium Smooth. Most scans were made with contrast administration (Iomeron 300). All scans were performed during inspiration breath hold.

### Image Processing

Center lumen lines (CLLs) of all aortoiliac trajectories and preoperative anatomical characteristics were obtained using the EVAR planning software Aquarius Intuition (TeraRecon, Inc, Foster City, CL, USA) with the incorporated workflow protocol, including manual optimization when necessary. The postoperative CLLs were created from the most proximal stent-ring of the body to the most distal stent-ring of the limb, resulting in 2 CLLs for each patient (right and left). To compare pre- and postoperative CLLs, the start and end points of the preoperative CLLs were determined based on anatomical landmarks, such as bifurcations, vessel branches, and calcifications on the first postoperative CT scan.

### Geometrical Parameters

The geometrical parameters length, curvature, torsion, and tortuosity index (TI) were calculated for all CLLs using previously described inhouse written algorithms in the Python programming language (version 3.7).^18^ First, the CLLs were sampled at 1 mm and filtered to obtain smooth differentiable curves. This was achieved using a Savitzky Golay filter of polynomial order 4 and window length 33, as confirmed by visual inspection. Length of the aortoiliac trajectory was defined as the length of the CLL. TI was calculated as the CLL length divided by the Euclidean distance. Both curvature (Equation 1)^18,19^ and torsion (Equation 2)^19^ were calculated for each CLL coordinate by numerical computation:

(1) $$\kappa =\frac{\sqrt{{\left(z^{\prime \prime }y^{\prime }-y^{\prime \prime }z^{\prime }\right)}^{2}+{\left(x^{\prime \prime }z^{\prime }-z^{\prime \prime }x^{\prime }\right)}^{2}+{\left(y^{\prime \prime }x^{\prime }-x^{\prime \prime }y^{\prime }\right)}^{2}}}{{\left({x^{\prime }}^{2}+{y^{\prime }}^{2}+{z^{\prime }}^{2}\right)}^{3/2}},$$

(2) $$\tau =\frac{x^{\prime \prime \prime }\left(y^{\prime }z^{\prime \prime }-y^{\prime \prime }z^{\prime }\right)+y^{\prime \prime \prime }\left(x^{\prime \prime }z^{\prime }-x^{\prime }z^{\prime \prime }\right)+z^{\prime \prime \prime }\left(x^{\prime }y^{\prime \prime }-x^{\prime \prime }y^{\prime }\right)}{{\left(y^{\prime }z^{\prime \prime }-y^{\prime \prime }z^{\prime }\right)}^{2}+{\left(x^{\prime \prime }z^{\prime }-x^{\prime }z^{\prime \prime }\right)}^{2}+{\left(x^{\prime }y^{\prime \prime }-x^{\prime \prime }y^{\prime }\right)}^{2}}$$

where *x*, *y*, and *z* are the Cartesian coordinates of the CLL, ′ is the first derivative, ″ the second derivative and ″′ the third derivative along the trajectory of the CLL. Curvature can be interpreted as the inverse of the radius of a circle fitted to the CLL for each CLL point, as previously described.^18^ Torsion can be interpreted as the extent to which curvature involves the third dimension, also for each CLL point. For example, a rollercoaster looping with a curvature of 1/”radius of the loop” ends not in the same plane as it starts, i.e. the loop involves a third dimension (torsion) and the further the start and end point of the this loop/curve are apart, the larger the torsion. For further analysis, the mean and maximal curvature and torsion values for each CLL were used. Since torsion values can also be negative, depending on torsion direction, the absolute torsion values of each CLL coordinate were used.

### Statistical Analysis

Normality of continuous variables was checked with a Shapiro-Wilk test. Continuous variables with a normal distribution were displayed as mean±standard deviation, continuous variables with a skewed distribution were displayed as median (interquartile range [IQR]) and categorical variables as number (percentage). The baseline characteristics of the PG and TE cohorts were compared with independent samples t-tests (normally distributed data) or Mann-Whitney U tests (skewed data) for continuous variables and Pearson’s Χ^2^ or Fisher Exact tests for categorical variables, as appropriate. Change (Δ) in the parameters was determined as the latter minus the former scan moment. Univariable Generalized Estimating Equations (GEE) were used to determine the potential univariate predictors for TE in a limb, while taking into account the interdependence of the right and left limbs within individual patients. Univariate predictors (p<0.10) and predictors suggested in literature (iliac calcification, distal limb diameter, and extension into the external iliac artery)^5,11^ were used as potential input variables for the multivariate GEE model for TE using backward elimination of variables according to likelihood ratio criteria (odd’s ratio [OR] and 95% confidence interval [CI]). Time-to-event data were analyzed as survival analysis using the Kaplan-Meier method.

Secondary analyses were performed of PG vs. LGO and PG vs. DLE, similar to the above described GEE method of PG vs. TE, to identify specific predictors for LGO and DLE, respectively. Furthermore, GEE were used to determine the predictive value of the changes in geometrical parameters during follow-up, that is, from the first postoperative CT to midterm follow-up.

Significance was set at p<0.05, except for the parameters resulting from the univariate analysis with a p-value <0.10. These were used as potential input parameters for the multivariate analysis. All statistical analyses were performed using IBM SPSS Statistics (version 25.0; IBM Corporation, Armonk, NY, USA).

## Results

**Figure 1.**
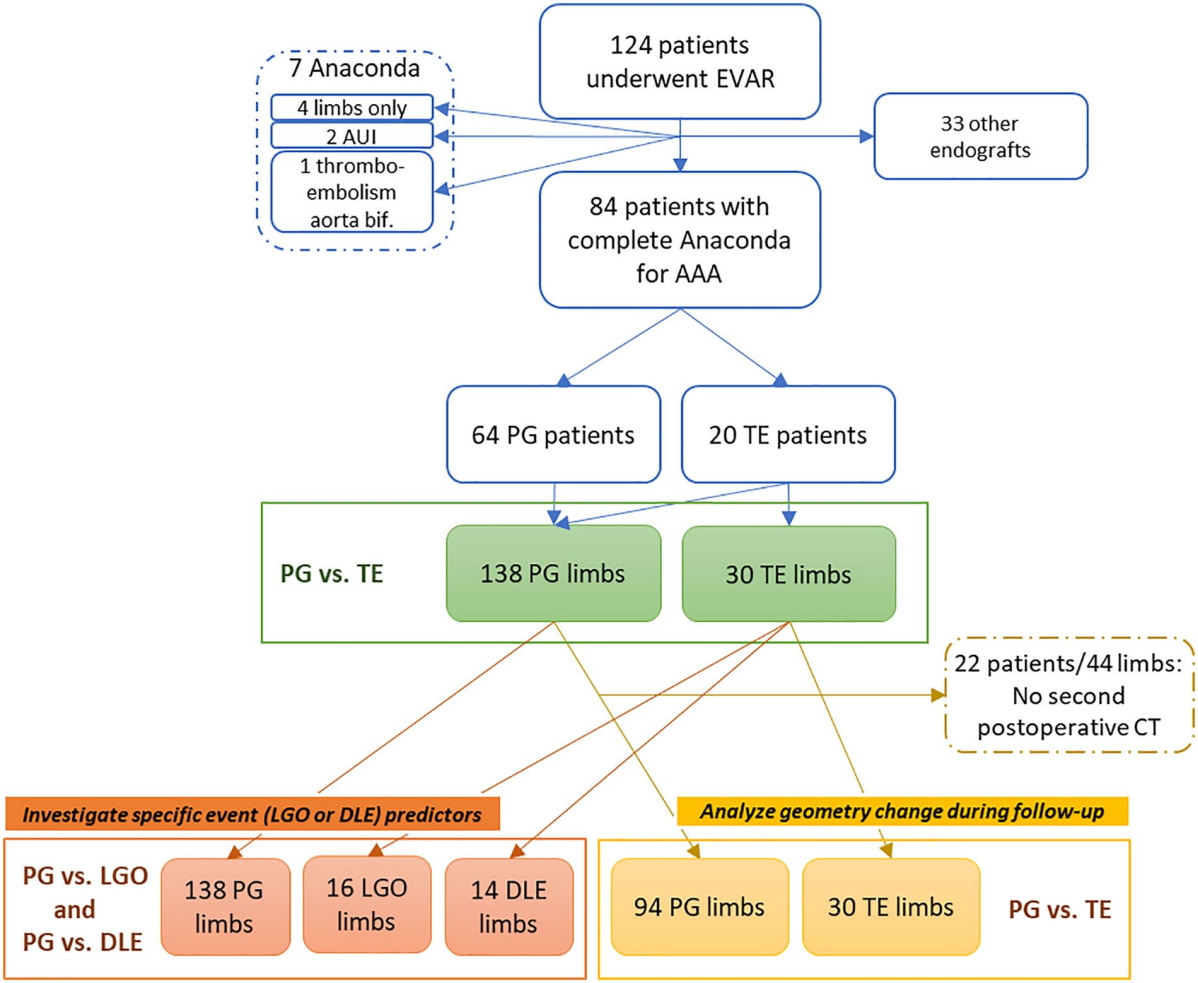
Flowchart of patient enrolment and categorization for analysis. The TE group was compared to the PG (i.e. without thromboembolic limb events) on limb parameters, including geometrical changes (green). Secondary analyses are investigation of the specific event types, so LGO versus PG group and DLE versus PG group (orange) and the change in geometrical parameters during follow-up was compared between the groups for the patients that had a second postoperative CT scan available (yellow). AAA, abdominal aortic aneurysm; AUI, aorto-uni-iliac device; bif, bifurcation; CT, computer tomography; DLE, distal limb embolization; EVAR, endovascular aneurysm repair; LGO, limb graft occlusion; PG, patent group; TE, thromboembolic event; vs., versus.

A total of 124 EVAR procedures were performed during the study period, of which 84 (mean age 74±8.3 years, 74 men) used the Anaconda stent-graft (Figure 1). Of these patients, 5 (5.9%) were treated outside of the instructions for use (neck angulation >90^o^, n=1; neck length <15 mm, n=2; native iliac diameter >21 mm, n=2). Sixteen patients were treated in acute setting (10 symptomatic and 6 ruptured AAAs). All Anaconda stent-grafts were second generation ONE-LOK systems and all were fully ballooned after placement with either a Constellation balloon (PanMedical, New Delhi, India) or Altosa balloon (Andratec GmbH, Koblenz, Germany). The postoperative anticoagulant schedule was single antiplatelet therapy, with a preference for clopidogrel, or the preoperative direct oral anticoagulants or Vitamin K antagonist was continued. Patient characteristics, anatomical characteristics and procedural parameters are summarized in Tables 1–3, respectively. All TE’s are listed in appendix A.

**Table 1.** Baseline Characteristics of the Cohort of AAA Patients Treated With an Anaconda Stent-Graft.

Characteristics	Total n=84	PG^a^ n=64	TE^b^ n=20	p*
*Patient Characteristics*				
Age—y	74±8	76±8	69±8	**<0.01**
Male gender	74 (88)	54 (84)	20 (100)	0.11
ASA grade				0.18
I	0 (0)	0 (0)	0 (0)	
II	24 (29)	15 (23)	9 (45)	
III	53 (63)	42 (66)	11 (55)	
IV	6 (7)	6 (9)	0 (0)	
V	1 (1)	1 (2)	0 (0)	
Body Mass Index	26±4	26±4	28±3	0.08
Diabetes mellitus	18 (21)	12 (19)	6 (30)	0.35
Smoking^c^	29 (35)	21 (33)	8 (40)	0.56
Hypertension				0.57
1 drug	47 (56)	37 (58)	10 (50)	
2 drugs	6 (7)	4 (6)	2 (10)	
≥3 drugs, uncontrolled	4 (5)	2 (3)	2 (10)	
Hyperlipidemia	57 (68)	43 (67)	14 (70)	0.81
Cardiac disease				0.79
Asymptomatic, MI	26 (31)	20 (31)	6 (30)	
Unstable angina, etc.	12 (14)	10 (16)	2 (10)	
Carotid disease^d^	9 (11)	7 (11)	2 (10)	1.00
Renal disease	7 (8)	5 (8)	2 (10)	0.67
Pulmonary disease				0.41
Mild	21 (2)	17 (27)	4 (20)	
Severe	4 (5)	2 (3)	2 (10)	
*Aneurysm Characteristics*				
Aortic neck				
Diameters—mm				
Proximal	23.1±2.6	23.0±2.8	23.4±2.0	0.54
Mid	23.0 (21.0, 26.0)	23.0 (21.0, 26.0)	23.5 (21.3, 25.8)	0.98
Distal	25.0 (22.3, 27.0)	25.0 (22.0, 26.8)	25.5 (23.0, 27.0)	0.53
Length—mm	35.0 (21.0, 43.0)	37.0 (20.5, 45.8)	27.5 (21.0, 39.5)	0.15
Angulation—degrees				
Α	17.0 (11.0, 27.3)	17.5 (11.0, 28.0)	15.5 (11.0, 24.0)	0.68
Β	35.0 (22.0, 46.8)	35.0 (22.0, 46.8)	32.5 (23.5, 47.0)	0.81
Circumferential thrombus - %	0.0 (0.0, 27.5)	0.0 (0.0, 20.0)	5.0 (0.0, 40.0)	0.19
Circumferential calcification - %	10.0 (0.0, 30.0)	10.0 (0.0, 30.0)	0.5 (0.0, 35.0)	0.28
Maximum AAA diameter –mm	60.5 (54.3, 68.0)	63.0 (54.0, 69.8)	58.0 (55.0, 61.8)	0.18

Normally distributed continuous data are presented as the mean±standard deviation; skewed distributed continuous data are presented as the median (inter quartile range [IQR]); categorical data are given as number (%).

Abbreviations: AAA, abdominal aortic aneurysm; ASA, American Society of Anesthesiologists; MI, myocardial infarction; PG, patent group; TE, thromboembolic event group.

a PG group consists of all patients with patent limbs, i.e. not diagnosed with a thromboembolic event.

b TE group consists of patients with a thromboembolic event (limb graft occlusion and/or distal limb embolism) in follow-up.

c Only includes current smokers.

d Transient ischemic attack, TIA and/or (ischemic) Cerebrovascular Accident, (i)CVA.

* p-value indicating a difference between PG and TE groups when <0.10, which are depicted bold in the table.

**Table 2.** Limb Characteristics of All Abdominal Aortic Aneurysm (AAA) Patients Treated With an Anaconda Stent-Graft.

Limb characteristics	Total n=168 limbs	PG^a^ n=138 limbs	TE^b^ n=30 limbs	p*	OR (95% CI)	B
*Iliac arteries*						
Diameter—mm						
CIA	14.0 (13.0, 17.0)	15.0 (13.0, 18.0)	14.0 (12.0, 16.3)	0.15	0.945 (0.876, 1.020)	−0.056
EIA	10.0 (9.0, 11.0)	10.0 (9.0, 11.0)	10.0 (9.0, 11.0)	0.95	1.005 (0.845, 1.196)	0.005
Circumferential thrombus CIA—%	0.0 (0.0, 10.0)	0.0 (0.0, 10.0)	0.0 (0.0, 20.0)	0.40	1.007 (0.990, 1.025)	0.007
Circumferential calcification CIA—%	30.0 (10.0, 50.0)	30.0 (10.0, 50.0)	20.0 (5.0, 40.0)	**0.03**	0.982 (0.966, 0.999)	−0.018
Mean curvature^c^—m^–1^	27.3±6.6	27.7±6.6	25.4±6.3	0.15	0.949 (0.883, 1.020)	−0.053
Maximal curvature^c^—m^–1^	90.9 (64.5, 114.3)	92.3 (71.0, 116.2)	87.9 (55.0, 112.3)	0.66	0.997 (0.983, 1.011)	−0.003
*Device characteristics*						
Body diameter^d^—mm	28.0 (25.0, 30.0)	28.0 (25.0, 30.0)	28.0 (25.0, 30.0)	0.68	1.032 (0.890, 1.196)	0.031
Bridge and/or extension	44 (26%)	36 (26%)	8 (27%)	0.96	1.030 (0.367, 2.891)	0.030
Length^e^—mm	138.2 (125.4, 155.8)	138.9 (125.2, 156.1)	132.3 (124.9, 155.1)	0.68	0.997 (0.980, 1.013)	−0.003
Distal limb diameter—mm	15.0 (13.5, 19.0)	15.0 (13.0, 19.0)	15.0 (15.0, 19.0)	0.99	0.999 (0.903, 1.106)	−0.001
Oversizing—%	15.0 (8.3, 22.5)	15.4 (7.1, 21.4)	18.5 (10.5, 25.0)	0.47	1.007 (0.989, 1.026)	0.007
Limb extension into EIA	23 (14%)	20 (14%)	3 (10%)	0.60	0.656 (0.136, 3.158)	−0.422

PG and TE were compared using Generalized Estimating Equations (GEE) analysis, also revealing odd’s ratio’s (OR). Normally distributed continuous data are presented as the mean±standard deviation; skewed distributed continuous data are presented as the median (inter quartile range [IQR]); categorical data are given as number (%).

Abbreviations: CIA, common iliac artery; EIA, external iliac artery; mm, millimeter; OR, odds ratio; PG, patent group; TE, thromboembolic event group; 95% CI, 95% confidence interval.

a PG group consists of all patent limbs, i.e. not diagnosed with a thromboembolic event.

b TE group consists of limbs with a thromboembolic event (limb thrombosis and/or limb embolism) in follow, up.

c Measured on the preoperative CT of the center lumen line (CLL) trajectory that will be covered by the stent, graft.

d The body diameter was included once for each limb (so twice in total)

e Measured on first postoperative CT as the CLL length from flow divider to last stent ring

* p-value indicating a difference between PG and TE groups when <0.10, which are depicted bold in the table.

**Table 3.** Changes (Δ, Latter Minus Former Scan Moment) in the Limb Geometry Parameter From Pre-to-Postoperative and During Follow-Up for All Limbs of the Abdominal Aortic Aneurysm (AAA) Patients Treated With an Anaconda Stent-Graft.

Changes in limb geometry	Total n=168 limbs	PG^a^ n=138 limbs	TE^b^ n=30 limbs	p*	OR (95% CI)	B
*Pre-to-postoperative change*						
Δ Length—mm	−14.4 (−23.3, −5.8)	−15.0 (−23.9, −6.5)	−10.8 (−19.5, −3.5)	0.67	1.007 (0.975–1.040)	0.007
Δ Mean curvature—m^−1^	−4.3±6.0	−4.8±6.0	−2.0±5.4	**0.03**	1.082 (1.007–1.164)	0.079
Δ Maximal curvature—m^−1^	−11.5±37.4	−11.8±37.4	−10.2±38.3	0.83	1.001 (0.990–1.012)	0.001
Δ Location maximal curvature—mm from proximal end of CLL	−15.3 (−76.4, 7.2)	−16.5 (−75.9, 8.1)	−8.7 (−89.4, 5.3)	0.67	1.001 (0.996–1.006)	0.001
Δ Mean torsion—m^−1^	2.6 (−14.1, 16.8)	2.0 (−14.4, 16.9)	3.2 (−12.3,16.1)	0.59	1.004 (0.989–1.020)	0.004
Δ Maximal torsion—m^−1^	−24.2 (−680.1, 650.0)	−43.8 (−721.0, 608.2)	240.6 (−409.1, 916.1)	0.24	1.000 (1.000–1.000)	9.592 *10^−5^
Δ Location maximal torsion—mm from proximal end of CLL	−16.7±86.0	−15.4±85.7	−23.0±88.4	0.66	0.999 (0.994–1.004)	−0.001
Δ TI	−0.071 (−0.112, −0.037)	−0.078 (−0.134, −0.038)	−0.052 (−0.093, −0.035)	**0.04**	1.295^c^ (1.009–1.662)	0.259^c^
*Midterm follow-up*	**Total** n=124 limbs	**PG**^a^ n=94 limbs	**TE** ^b^ **n=30 limbs**			
Δ Length—mm	−1.3 (−5.6, 2.2)	−0.6 (−4.3, 2.4)	−4.5 (−8.4, −0.2)	**0.08**	0.947 (0.892–1.006)	−0.054
Δ Mean curvature—m^−1^	1.7 (−1.4, 4.0)	1.7 (−1.5, 4.0)	1.3 (−1.4, 4.0)	0.51	0.970 (0.885–1063)	−0.031
Δ Maximal curvature—m^−1^	−0.6 (−22.7, 18.1)	−2.5 (−23.0, 19.7)	3.3 (−17.2, 15.7)	0.89	0.999 (0.989–1.010)	−0.001
Δ Location maximal curvature—mm from proximal end of CLL	2.3 (−22.7, 48.4)	0.3 (−26.5, 33.1)	8.4 (−8.4, 74.4)	0.24	1.003 (0.998–1.007)	0.003
Δ Mean torsion—m^−1^	0.1±19.9	−0.8±18.7	3.0± 23.4	0.39	1.010 (0.988–1.033)	0.010
Δ Maximal torsion—m^−1^	140.2 (−815.3, 1049.4)	168.3 (−777.6, 1082.1)	−226.8 (−964.1, 551.5)	0.57	1.000 (1.000–1.000)	8.324 *10^−5^
Δ Location maximal torsion—mm from proximal end of CLL	−5.1±81.6	−12.1±82.0	16.9±77.8	**0.10**	1.005 (0.999–1.010)	0.004
Δ TI	0.017 (−0.001, 0.045)	0.017 (−0.002, 0.050)	0.015 (0.002, 0.034)	0.16	0.759^c^ (0.517–1.113)	−0.276^c^

PG and TE were compared using Generalized Estimating Equations (GEE) analysis, also revealing odd’s ratio’s (OR). Normally distributed continuous data are presented as the mean±standard deviation; skewed distributed continuous data are presented as the median (inter quartile range [IQR]); categorical data are given as number (%). Variables that differ between PG and TE with a p<0.10 were used for multivariate GEE and depicted bold in the table.

Abbreviations: CLL, center lumen line; mm, millimeter; OR, odds ratio; PG, patent group; TE, thromboembolic event group; TI, tortuosity index; 95% CI, 95% confidence interval.

a PG group consists of all patent limbs, i.e. not diagnosed with a thromboembolic event.

b TE group consists of limbs with follow-up thromboembolic events (limb graft thrombosis and/or distal embolization).

c The OR(95% CI) and B resulting from the GEE for TI were obtained after rearranging the variable to make steps of .05 instead of 1, considering the magnitude of TI.

* p-value indicating a difference between PG and TE groups when <0.05.

**Figure 2.**
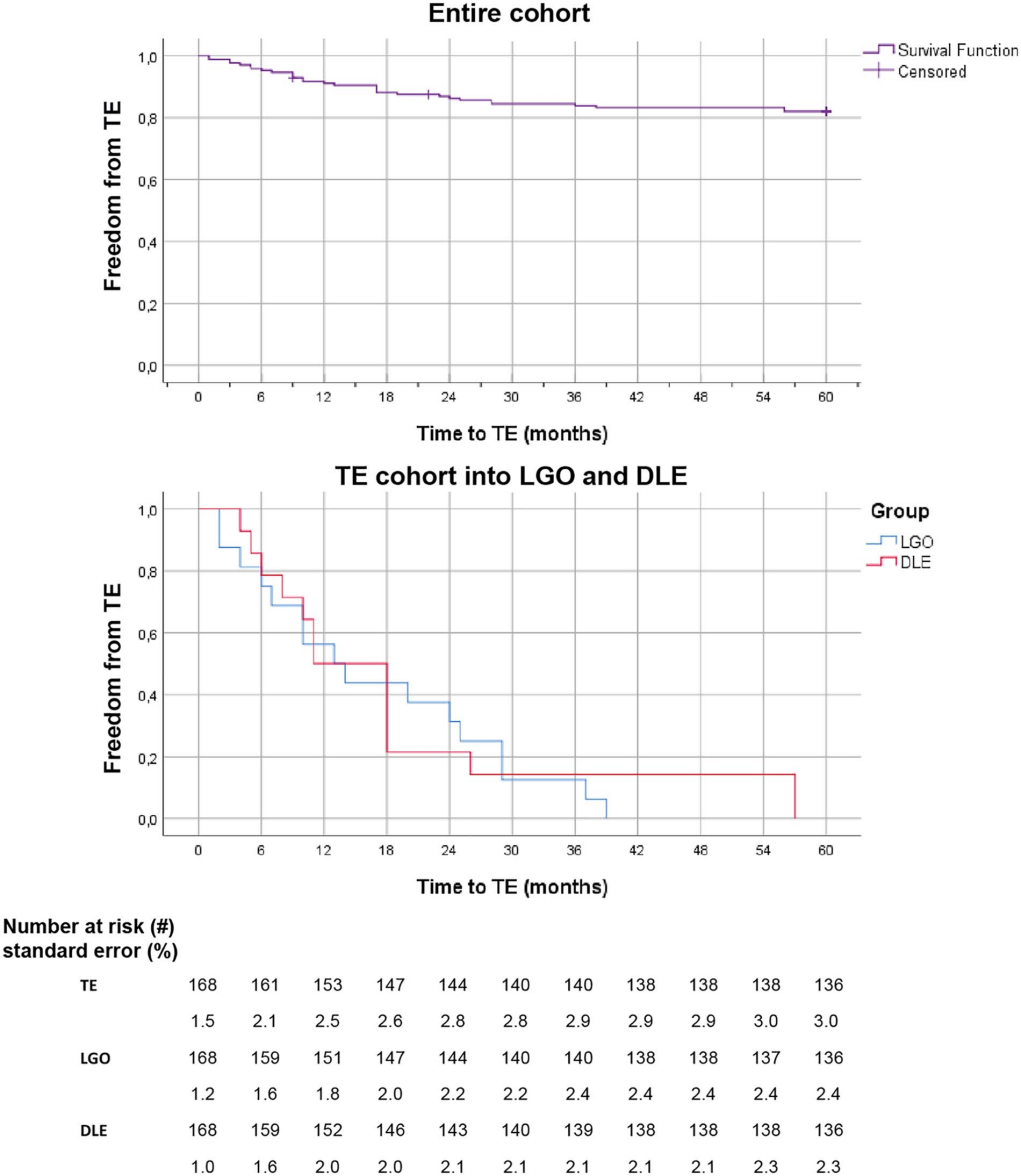
Survival analysis with Kaplan Meier plots for freedom from a TE within the entire cohort (n=84, purple) and separately for LGO (blue) and DLE (red), all in months after endovascular aneurysm repair (EVAR). DLE, distal limb embolization; LGO, limb graft occlusion; TE, thromboembolic event.

TE was observed in 20 patients (30 limbs, 17.8%); LGO in 10 patients (14 limbs, 8.3%) and DLE in 10 patients (16 limbs, 9.5%) during a mean follow-up of 39±15 months, (PG: 40±15 months; TE: 38±17 months, ns), see Figure 2. A summary of the TE patients, including event side and treatment, can be found in appendix A. Age was the only statistically significant difference in baseline characteristics between the groups: the PG cohort is on average 7 years older than the TE cohort. The LGO cohort had a higher level of circumferential aortic neck thrombus than the PG cohort (p=0.05) and the DLE cohort was younger (p<0.01) and had a lower ASA grade (p=0.03) than the PG cohort (Appendix B).

The median time between surgery and the first postoperative scan was 77 (12-93) days (PG: 78 [13-94] days; TE: 50 (7-91) days, ns). Twenty-two patients did not have a second postoperative CT available. Therefore, midterm follow-up geometrical changes were determined for 62 patients (124 limbs: 94 PG and 30 TE, with 16 LGO and 14 DLE) with the time between surgery and the second postoperative scan 354 (221-538) days (PG: 350 [122-619] days; TE 366 [265-401] days, ns).

### Univariate Analysis

*Pre-to-post implantation geometrical changes*: Univariate analysis identified 3 factors related to the development of TE: The odds for a TE were lowered with a larger degree of circumferential CIA calcification (OR: 0.98, 95% CI: 0.97–1.00, p=0.03), larger decrease in mean curvature from pre-to-post implant (OR: 1.08, 95% CI: 1.01–1.16, p=0.03), and larger decrease in TI from pre-to-post implant (steps of 0.05: OR: 1.30, 95% CI: 1.01–1.66, p=0.04), Tables 2 and 3.

One univariate predictor for LGO was identified: the caudal relocation of the curvature maximum from pre-to-post EVAR (OR: 1.01, 95% CI: 1.00–1.01, p=0.04, appendix B). Univariate predictors for DLE were smaller CIA diameter (OR: 0.87, 95% CI: 0.76–0.99, p=0.04), less circumferential CIA calcification (OR: 0.97, 95% CI: 0.94–0.99, p=0.02), smaller mean and maximal curvature of the preoperative aortoiliac trajectory (OR: 0.86, 95% CI: 0.79–0.94, p<0.01 and OR: 0.97, 95% CI: 0.95–1.00, p=0.03, respectively), and smaller pre-to-post implant decrease in mean curvature (OR: 1.11, 95% CI: 1.02–1.21, p=0.02, Appendix B).

*Midterm geometrical changes*: Two geometrical parameter changes were identified as univariate midterm follow-up predictors for TE: Length decrease, that is, shortening (OR: 0.95, 95% CI: 0.89–1.01, p=0.08) and cranial relocation of the torsion maximum (OR: 1.005, 95% CI: 0.99–1.01, p=0.10), Table 3. Predictors for a DLE were the downstream relocation of the curvature maximum (OR: 1.01, 95% CI: 1.00–1.01, p=0.01) and of the torsion maximum (OR: 1.01, 95% CI: 1.00–1.01, p=0.02). No midterm geometrical parameter changes were identified as LGO predictor, Table 3 and Appendix B.

### Multivariate Analysis

The prediction model for TE included circumferential CIA calcification (B -0.023, OR: 0.977, 95% CI: 0.960–0.995, p=0.01) and pre-to-post EVAR change in mean curvature (B -0.106, OR: 1.112, 95% CI: 1.025–1.207, p=0.01). Note that the mean curvature changes are negative and thus the mean curvature decreases in both groups, though the decrease is stronger in the PG cohort. A model with circumferential CIA calcification (B -0.027, OR: 0.974, 95% CI: 0.956–0.992, p=0.01) and midterm change in aortoiliac length (B -0.058, OR: 0.944, 95% CI: 0.895–0.995, p=0.03) seemed strong as well (based on the likelihood ratio criteria), though that was based on only the 62 patients that had a midterm CT. The pre-to-postoperative decrease in TI and postoperative length decrease and torsion maximum relocation were excluded as predictors. Furthermore, forcing any of the potential predictors proposed in literature (distal limb diameter, limb extension into the EIA and limb bridging and/or extension) did not lead to improvement of the model.

For LGO prediction, multivariate analyses did not reveal an additional predictor to pre-to-postoperative change in location of maximal curvature. For DLE prediction, the optimal model combined preoperative mean curvature (OR: 0.839, 95% CI: 0.765–0.919, p<0.01), circumferential CIA calcification (OR: 0.958, 95% CI: 0.924–0.994, p=0.02), and CIA diameter (OR: 0.835, 95% CI: 0.715–0.976, p=0.02). Forcing any of the literature proposed predictors into the models did not improve the DLE model.

## Discussion

This study showed that the geometric parameters curvature and TI of the aortoiliac trajectory are altered up to 1 year after EVAR. The odds of TE development drop with decreasing mean curvature and TI of the stented aorto-iliac trajectory from pre-to-post implant and with a higher pre-operative percentage of circumferential CIA calcification. Decrease in length during the first year post-EVAR seems to also increase the odds for TE development. Since the aortoiliac trajectory is straightened during EVAR by the stiff guidewire, the stent-graft is deployed in this straightened environment. It is presumed that after EVAR the more calcified CIAs of the PG patients remain in this straightened position and thereby actuate the larger decrease in curvature and TI. Hence, these limbs could experience a more straight blood flow path. The less calcified and thereby more flexible CIAs of the TE patients may tend to go back to their original, tortuous state after removal of the stiff guidewire and could therefore show a smaller decrease in curvature and TI after EVAR.

**Figure 3.**
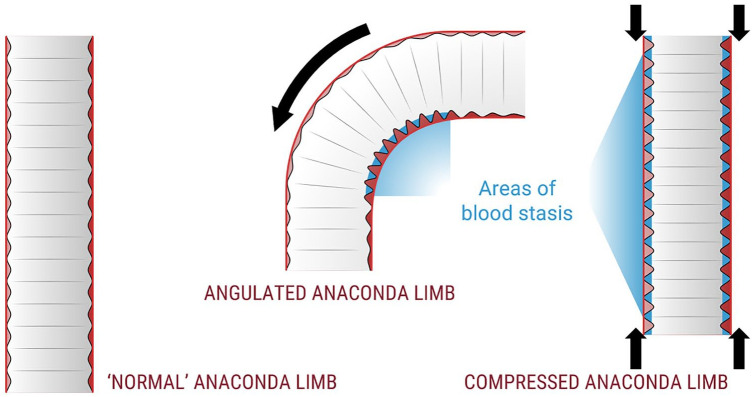
The Concertina effect hypothesis in the Anaconda stent-graft: in straightened Anaconda limbs the walls are relatively straight, but with increasing angulation and/or decreasing limb length, the separate nitinol rings are pressed together, inducing infolding of the graft fabric into the lumen. These plications may induce areas of blood stasis (blue) that are suspect for thrombus formation.

As the aortoiliac trajectories of the TE patients were straightened when the stent-graft is placed and go back to their original state after removal of the stiff guidewire, the induced curves in the stent graft limbs are thought to be more prone to the Concertina effect (Figure 3). The Concertina effect implies that in tortuous and/or shortened (Anaconda) stent-graft limbs the neighboring nitinol stent-rings come closer together, which may cause inward folding of the graft-fabric between these stent-rings. If these resulting plications reduce the blood flow and wall shear stress, thrombus formation may be promoted.^20^ The results of a previous prospective study conducting in depth analysis of 15 Anaconda patients support the Concertina effect hypothesis as a correlation was found between the decreasing distances between stent rings and decrease in limb length and increase in curvature.^18^ Moreover, the 2 patients in that cohort who developed limb occlusion showed a severe postoperative increase in curvature and small distances between the individual stent rings. Furthermore, aortoiliac remodeling during the first year post-EVAR reduces the aneurysm size both in transverse and longitudinal direction and thereby shortens this trajectory, which could further attribute to the Concertina effect. Hence, the Concertina effect may be elemental to the elevated LGO-rate in Anaconda limbs,^5,14,15^ which is also seen in the present cohort where 16 of the 168 limbs occluded (9.5%) on mid-term.

EVAR limb geometry (changes) have been only partially investigated. Tasso et al^21^ investigated the aortoiliac geometry and hemodynamics of Endurant and Excluder stent-grafts. They observed a higher curvature in post-EVAR patients than in healthy subjects, especially in the limbs. Moreover, they found a relation between the maximal curvature and torsion values and the percentage of recirculating blood volume computed with computational fluid dynamics (CFD), which indicates that increased curvature and torsion along the aortoiliac trajectory elevate the risk of thrombus formation.^20^ These findings complement the present results and substantiate the Concertina effect hypothesis. Further investigation into the presence of the Concertina effect in Anaconda and other EVAR-stent-grafts may be conducted in the field of ultrasound particle image velocimetry (ePIV)^22^ or computational fluid dynamics (CFD).^21^

Similar to the findings of Cochennec et al,^23^ the patients with a TE event during follow-up were younger than those with patent limbs during follow-up, although we have no explanation for this finding. However, it emphasizes the importance of considering open repair in younger AAA patients. The previously established relation between limb occlusion and distal limb diameter^5^ and limb extensions^2^ was not found in the present study. This may be attributed to the ballooning of the stent-graft that was performed in each patient, which improves clinical outcomes.^24,25^ Contrasting to previous studies by Mantas et al^14^ and Carroccio et al^15^ who found CIA calcification to be a predisposing factor for limb thrombosis, the present study revealed it to be a protective factor. An explanation for this may be found in the study methodologies. Mantas et al^14^ categorized the calcification in <50% and ≥50% calcification, while we used the circumferential calcification degree (%) as a continuous variable. In addition, only 12 of the 72 reviewed stentgrafts were Anaconda’s. Also, the study of Carroccio et al^15^ was published in 2002 and reviewed other (and older generation) devices than the Anaconda. Our hypothesis is that in a stiff calcified iliac trajectory the Anaconda stays more stretched which prohibits the Concertina effect.

The risk of LGO was separately investigated and seemed to increase with caudal relocation of maximal curvature, which could hypothetically induce local flow disturbances. The risk for a DLE was lowered by pre-to-post implant decrease in mean curvature, larger CIA diameter, higher degree of CIA calcification and larger preoperative curvature of the aortoiliac trajectory. A less straightened aortoiliac trajectory, that is, smaller decrease in mean curvature, can be related to the above mentioned Concertina effect. A larger CIA diameter may provide less disturbed outflow and thereby a higher patency rate.^5,6,15,23^ The difference in parameters influencing LGO and DLE may be due to the number of events and the degree of flow disturbance. Caudal relocation of the maximal curvature may have a more local influence on the blood flow patterns and thereby induce the local problem of LGO, while a smaller CIA diameter, smaller preoperative curvature with a smaller curvature decrease and less CIA calcification may only alter the blood flow to the point where blood starts to clot and thromboembolisms are formed, but not to the extent that the entire limb gets occluded. However, the exact mechanisms behind these observations and hypotheses need further investigation.

The TEs and the performed treatments, listed in appendix A, reveal that realigning the limbs with Gore Excluder limbs avoids re-TE in 9 of the 10 limbs that underwent this TE-treatment. This is in line with the recent publication of Marino et al.^26^ Although relining may aid in future durability of the device, note should be made of the additional costs that come with relining. Relining had limited to no influence on the postoperative changes calculated in this study.

Although curvature is a relatively novel parameter for aorto-iliac geometry quantification, curvature has been investigated as a predictor for complications after EVAR before.^18,19,27–31^ However, to fully describe the geometrics of a 3D CLL, torsion should be considered as well. Torsion describes the extent to which the induced curvature involves the third dimension. Even though this parameter is very sensitive to the smallest inaccuracies in the CLL, its potential in predicting complications after EVAR is promising.^19,21^ The retrospective nature of the study may be considered a limitation, though it does provide useful insights in this specific device.

## Conclusion

The present study confirms that treatment of infrarenal AAA with an Anaconda stent-graft is related to a relatively high TE rate in the years following implantation. This study revealed that the TE-risk is lowered by a higher degree of pre-intervention circumferential CIA calcification and a larger decrease in curvature and TI pre-to-post EVAR. These findings support the Concertina effect hypothesis, which may also be related to the high angulation cohort which Anaconda devices are used to treat. Further investigation into the mechanisms behind this effect and consequently targeting stent-grafts design and treatment improvements are pivotal.

## Appendix A Summary of Events

Table A1 shows the TE’s (LGO and DLE) and the treatments of the individual patients.

**Table A1.** Summary of Thromboembolic Events of the Anaconda Patients, Divided in LGO Group and DLE Groups.

Patient ID	Number of events	Group	Side of event	Type of event	Treatment	Months after EVAR
1	6	LGO	R	LGO	Embolectomy + realigning with Wallstent 10 mm	1.5 (43 days)
			L	LGO	Embolectomy	13
			L	LGO	Embolectomy + PTA	19
			L	LGO	Realignment with 2 Gore Excluder limbs	22
			R	LGO	Thrombolysis + PTA + Wallstent in EIA	24
			R	LGO	Gore excluder limb + Viabahn stent	25
2	3	LGO	R	LGO	Thrombolysis	6
			Both	LGO	Realignment with Gore Excluder limbs	9
			L	DLE	PTA popliteal artery, truncus tibioperonealis, posterior and anterior tibial artery	13
3	4	LGO	L	DLE	Thrombolysis + PTA popliteal artery, posterior tibial artery	12
			R	LGO	Thrombolysis + PTA + realignment 2 Gore Excluder limbs	19
			L	DLE	Thrombolysis	24
			L	LGO	Realignment 2 Gore Excluder limbs	30
4	3	LGO	L	DLE	Embolectomy + PTA	9
			R	LGO	Realignment both limbs with Gore Excluder limbs	37
			L	LGO	Extend Gore limb to proximal + PTA	38
5	1	LGO	R	LGO	PTA + bare metal stent in transition body to limb	2
6	4	LGO	Both	LGO	Embolectomy	28
			R	LGO	Thrombolysis	31
			R	LGO	Thrombolysis	40
			R	LGO	Realignment both limbs with Gore Excluder limbs	41
7	2	LGO	L	LGO	PTA + stent	3
			R	LGO	Thrombolysis + realignment Gore Excluder limb	24
8	1	LGO	L	LGO	Thrombolysis + realignment Gore Excluder limb	5
9	1	LGO	R	LGO	Realignment Gore Excluder limbs	38
10	1	LGO	L	LGO	Embolectomy + realignment Gore Excluder limbs	24
11	4	DLE	L	DLE	Thrombolysis popliteal artery	9
			L	DLE	Thrombolysis + embolectomy popliteal artery	13
			L	DLE	Thrombolysis	16
			R	DLE	Thrombolysis	17
12	1	DLE	R	DLE	Thrombolysis + PTA + stent	7
13	2	DLE	R	DLE	Thrombolysis	5
			R	DLE	Thrombolysis	10
14	1	DLE	Both	DLE	Embolectomy + bypass	17
15	1	DLE	L	DLE	PTA	4
16	1	DLE	L	DLE	Conservative	3
17	1	DLE	L	DLE	Realignment with Gore Excluder limbs + embolectomy popliteal artery	10
18	1	DLE	Both	DLE	Realignment with Gore Excluder limbs + embolectomy popliteal artery	56
19	1	DLE	L	DLE	PTA	25
20	1	DLE	R	DLE	PTA	17

Abbreviations: DLE, distal limb embolization; EIA, external iliac artery; EVAR, endovascular aneurysm repair; L, left limb; LGO, limb graft occlusion; PTA, percutaneous transluminal angioplasty; R, right limb.

## Appendix B—Univariate Analysis of LGO Versus PG Group and DLE Versus PG Group

The patient characteristics, limb characteristics and changes in limb geometry parameters of PG, LGO and DLE are shown in Table B1, Table B2 and Table B3, respectively.

**Table B1.** Patient and Aneurysm Characteristics, of the PG, LGO Group and DLE Group.

Characteristics	PG^a^ n=64	LGO^b^ n=10	p_PGvsLGO_*	DLE^b^ n=10	p_PGvsDLE_*
*Patient Characteristics*					
Age—y	76±8	72±6	0.20	66±8	**<0.01**
Male gender	54 (84%)	10 (100%)	0.18	10 (100%)	0.18
ASA grade			0.71		**0.03**
I	0 (0%)	0 (0%)		0 (0%)	
II	15 (23%)	2 (20%)		7 (70%)	
III	42 (66%)	8 (80%)		3 (30%)	
IV	6 (9%)	0 (0%)		0 (0%)	
V	1 (2%)	0 (0%)		0 (0%)	
Body Mass Index	26±4	29±3	0.07	27±3	0.47
Diabetes mellitus	12 (19%)	3 (30%)	0.41	3 (30%)	0.41
Smoking^c^	21 (33%)	5 (50%)	0.29	3 (30%)	0.86
Hypertension			0.32		0.65
1 drug	37 (58%)	4 (40%)		6 (60%)	
2 drugs	4 (6%)	2 (20%)		0 (0%)	
≥3 drugs, uncontrolled	2 (3%)	1 (10%)		1 (10%)	
Hyperlipidemia	43 (67%)	8 (80%)	0.42	6 (60%)	0.66
Cardiac disease			0.21		0.07
Asymptomatic, MI	20 (31%)	6 (60%)		0 (0%)	
Unstable angina, etc.	10 (16%)	1 (10%)		1 (10%)	
Carotid disease^d^	7 (11%)	1 (10%)	0.93	1 (10%)	0.93
Renal disease	5 (8%)	0 (0%)	0.60	2 (20%)	0.44
Pulmonary disease			0.84		0.06
Mild	17 (27%)	3 (30%)		1 (10%)	
Severe	2 (3%)	0 (0%)		2 (20%)	
*Aneurysm Characteristics*					
Aortic neck					
Diameters—mm					
Proximal	23.0±2.8	23.6±2.3	0.56	23.2±1.8	0.82
Mid	23.0 (21.0, 26.0)	24.0 (22.0, 24.5)	0.70	22.5 (22.0, 25.3)	0.67
Distal	25.0 (22.0, 26.8)	25.5 (22.8, 27.0)	0.64	25.5 (22.8, 27.0)	0.63
Length—mm	37.0 (20.5, 45.8)	28.0 (23.0, 41.8)	0.64	24.0 (20.3, 35.8)	0.09
Angulation—degrees					
α	17.5 (11.0, 28.0)	17.0 (8.8, 25.3)	0.56	15.5 (11.0, 25.0)	0.98
Β	35.0 (22.0, 46.8)	38 (27.5, 53.5)	0.50	28.5 (21.8, 35.5)	0.30
Circumferential thrombus—%	0.0 (0.0, 20.0)	10.0 (0.0, 47.5)	**0.05**	0.0 (0.0, 40.0)	0.98
Circumferential calcification - %	10.0 (0.0, 30.0)	5.0 (0.0, 42.5)	0.69	0.5 (0.0, 17.5)	0.22
Maximum AAA diameter—mm	63.0 (54.0, 69.8)	60.0 (53.3, 61.8)	0.23	56.5 (54.8, 64.0)	0.41
*Follow, up time—months*	40±15	41±18	0.86	36±17	0.50

Continuous data with normal distribution are presented as the mean±standard deviation; continuous data with skewed distribution are presented as the median (inter quartile range [IQR]); categorical data are given as number (%).

Abbreviations: AAA, abdominal aortic aneurysm; ASA, American Society of Anesthesiologists; DLE, distal limb embolization; LGO, limb graft occlusion; MI, myocardial infarction; PG, patent group.

a PG group consists of all patients with patent limbs, i.e. not diagnosed with a thromboembolic event.

b LGO group consists of patients with a limb thrombosis in follow, up; DLE group consists of patients with a limb embolism in follow, up.

c Only includes current smokers.

d Transient ischemic attack, TIA and/or (ischemic) Cerebrovascular Accident, (i)CVA.

* p, value indicating a difference between PG and LGO or DLE groups when <0.05, which are depicted bold in the table.

**Table B2.** Limb Characteristics Compared Between the PG, the LGO Group and the DLE Group.

Limb characteristics	PG^a^ n=138 limbs	LGO^b^ n=16 limbs	p _PGvsLGO_*	Odds ratio _PGvsLGO_ (95% CI)	B _PGvsLGO_	DLE^b^ n=14 limbs	p _PGvsDLE_*	Odds ratio (95% CI)	B _PGvsDLE_
*Iliac arteries*									
Diameter—mm									
CIA	15.0 (13.0, 18.0)	14.0 (12.0, 19.0)	0.49	0.976 (0.910–1.045)	−0.025	14.0 (12.0, 14.3)	**0.04**	0.869 (0.761–0.993)	−0.140
EIA	10.0 (9.0, 11.0)	10.0 (9.0, 10.8)	0.97	1.004 (0.805–1.253)	0.004	10.0 (9.0, 11.3)	0.95	1.006 (0.822–1.232)	0.006
Circumferential thrombus CIA - %	0.0 (0.0, 10.0)	0.0 (0.0, 47.5)	0.12	1.016 (0.996–1.036)	0.018	0.0 (0.0, 12.5)	0.39	0.990 (0.967–1.013)	−0.010
Circumferential calcification CIA - %	30.0 (10.0, 50.0)	30.0 (2.5, 50.0)	0.30	0.989 (0.969–1.010)	−0.011	15.0 (5.0, 25.0)	**0.03**	0.973 (0.950–0.997)	−0.027
Mean curvature^c^ —m^−1^	27.7±6.6	28.5±6.0	0.65	1.020 (0.936–1.112)	0.020	21.9±4.5	**<0.01**	0.863 (0.792–0.941)	−0.147
Maximal curvature^c^ —m^−1^	92.3 (71.0, 116.2)	101.5 (77.0, 116.6)	0.13	1.009 (0.997–1.021)	0.009	57.9 (46.6, 89.6)	**0.03**	0.973 (0.949–0.997)	−0.028
*Device characteristics*									
Body diameter^d^—mm	28.0 (25.0, 30.0)	30.0 (25.0, 32.0)	0.40	1.103 (0.877–1.387)	0.098	28.0 (25.0, 28.5)	0.48	0.952 (0.831–1.090)	−0.049
Bridge and/or extension	36 (26%)	7 (44%)	0.20	2.204 (0.662–7.334)	0.790	1 (7%)	0.15	0.218 (0.028–1.721)	−1.523
Length^e^—mm	138.9 (125.2, 156.1)	147.0 (126.1, 165.8)	0.90	1.001 (0.980–1.023)	0.001	130.4 (121.2, 142.4)	0.38	0.990 (0.968–1.013)	−0.010
Distal diameter—mm	15.0 (13.0, 19.0)	16.0 (13.5, 21.0)	0.72	1.026 (0.892-1.180)	0.026	15.0 (15.0, 17.0)	0.55	0.965 (0.860-1.084)	−0.035
Oversizing - %	15.4 (7.1, 21.4)	17.1 (10.8, 22.5)	0.33	1.010 (0.990–1.032)	0.010	19.6 (7.1, 25.0)	0.91	1.001 (0.980–1.023)	0.001
Limb extension into EIA	20 (14%)	3 (19%)	0.71	1.362 (0.268–6.919)	0.309	0 (0%)	*Not calculatable due to all in CIA in DLE group*		

Continuous data with normal distribution are presented as the mean±standard deviation; continuous data with skewed distribution are presented as the median (inter quartile range [IQR]); categorical data are given as number (%).

Abbreviations: CIA, common iliac artery; DLE, distal limb embolization; EIA, external iliac artery; LGO, limb graft occlusion; MI, myocardial infarction; mm, millimeter; PG, patent group; 95% CI, 95% confidence interval.

a PG group consists of all patent limbs, i.e. not diagnosed with a thromboembolic event.

b LGO group consists of limbs with a limb thrombosis in follow-up; DLE group consists of limbs with a limb embolism in follow-up.

c Measured on the preoperative CT of the center lumen line (CLL) trajectory that will be covered by the stent-graft.

d The body diameter was included once for each limb (so twice in total).

e Measured on first postoperative CT as the CLL length from flow divider to last stent ring.

* p-value indicating a difference between PG and LGO or DLE groups when <0.05, which are depicted bold in the table.

**Table B3.** Changes in the Limb Geometry Parameters From Pre- to Postoperative and During Follow-Up, of the PG, the LGO Group and DLE Group.

*Pre to postoperative change*	**PG**^a^ n=138 limbs	**LGO**^b^ n=16 limbs	**p**_PGvsLGO_ *	**Odds ratio**_PGvsLGO_ (95% CI)	**B _PGvsLGO_**	**DLE**^b^ n=14 limbs	**p**_PGvsDLE_ *	**Odds ratio**_PGvsDLE_ **(95% CI)**	**B_PGvsDLE_**
Δ Length—mm	−15.0 (−23.9, −6.5)	−18.1 (−29.2, −4.4)	0.56	0.988 (0.951–1.027)	−0.012	−8.3 (−14.7, −3.2)	0.11	1.033 (0.993–1.074)	0.033
Δ Mean curvature—m^−1^	−4.8±6.0	−3.0±5.3	0.24	1.052 (0.966–1.146)	0.051	−0.9±5.5	**0.02**	1.110 (1.015–1.214)	0.104
Δ Maximal curvature—m^−1^	−11.8±37.4	−17.9±81.5	0.54	0.996 (0.983–1.009)	−0.004	−1.4±33.1	0.31	1.008 (0.992–1.024)	0.008
Δ Location maximal curvature—mm from proximal end of CLL	−16.5 (−75.9, 8.1)	−5.3 (−19.3, 16.0)	**0.04**	1.006 (1.000–1.012)	0.006	−81.0 (−103.4, −4.4)	0.09	0.995 (0.989–1.001)	−0.005
Δ Mean torsion—m^−1^	2.0 (−14.4, 16.9)	1.2 (−20.3, 16.2)	0.87	0.998 (0.979–1.018)	−0.002	4.5 (−3.9, 16.8)	0.26	1.011 (0.991–1.032	0.011
Δ Maximal torsion—m^−1^	−43.8 (−721.0, 608.2)	130.5 (−722.9, 1189.3)	0.47	1.000 (1.000–1.000)	7.012* 10^−5^	364.1 (−409.0, 916.1)	0.17	1.000 (1.000–1.000)	0.000
Δ Location maximal torsion—mm from proximal end of CLL	−15.4±85.7	−10.9±104.8	0.85	1.001 (0.994–1.007)	0.001	−36.8±66.2	0.34	0.997 (0.991–1.003)	−0.003
Δ TI	−0.078 (−0.134, −0.038)	−0.059 (−0.098, −0.040)	0.12	1.221^c^ (0.948–1.572)	0.200^c^	−0.041 (−0.076, −0.020)	0.08	1.344^c^ (0.963–1.876)	0.296^c^
*Midterm follow-up*									
Δ Length—mm	−0.6 (−4.3, 2.4)	−2.3 (−13.2, 1.1)	0.08	0.949 (0.894–1.007)	−0.053	−4.9 (−7.8, −0.2)	0.10	0.961 (0.916–1.007)	−0.040
Δ Mean curvature—m^−1^	1.7 (−1.5, 4.0)	1.3 (−1.8, 3.9)	0.34	0.944 (0.838–1.063)	−0.058	1.5 (−0.9, 4.6)	0.98	1.002 (0.902–1.113)	0.002
Δ Maximal curvature—m^−1^	−2.5 (−23.0, 19.7)	−4.6 (−26.7, 17.8)	0.51	0.995 (0.982–1.009)	−0.005	−4.1 (−8.1, 12.4)	0.37	1.004 (0.995–1.014)	0.004
Δ Location maximal curvature—mm from proximal end of CLL	0.3 (−26.5, 33.1)	−2.8 (−17.7, 13.5)	0.20	0.998 (0.994–1.001)	−0.002	71.5 (4.4, 95.8)	**0.01**	1.008 (1.002–1.014)	0.008
Δ Mean torsion—m^−1^	−0.8±18.7	4.7±23.2	0.35	1.015 (0.984–1.048)	0.015	1.0±24.2	0.77	1.005 (0.973–1.038)	0.005
Δ Maximal torsion—m^−1^	138.3±1 624.5	−288.6±1 830.4	0.41	1.000 (0.999 −1.000)	0.000	124.5±2027.3	0.98	1.000 (1.000 −1.000)	−5.000* 10^−6^
Δ Location maximal torsion—mm from proximal end of CLL	−12.1±82.0	0.4±74.1	0.59	1.002 (0.995–1.009)	0.002	35.9±80.3	**0.02**	1.007 (1.001–1.013)	0.007
Δ TI	0.017 (−0.002, 0.050)	0.013 (0.005, 0.038)	0.31	0.760^c^ (0.449–1.287)	−0.274^c^	0.015 (0.001, 0.023)	0.24	0.764^c^ (0.487–1.199)	−0.269^c^

PG and LGO or DLE were compared using Generalized Estimating Equations (GEE) analysis, also revealing odd’s ratio’s (OR). Normally distributed continuous data are presented as the mean±standard deviation; skewed distributed continuous data are presented as the median (inter quartile range [IQR]); categorical data are given as number (%). Variables that differ between PG and LGO or DLE with a p<0.10 were used for multivariate GEE.

Abbreviations: CLL, center lumen line; DLE, distal limb embolization; LGO, limb graft occlusion; MI, myocardial infarction; mm, millimeter; PG, patent group; TI, tortuosity index; 95% CI, 95% confidence interval.

a PG group consists of all patent limbs, i.e., not diagnosed with a thromboembolic event.

b LGO group consists of limbs with a limb thrombosis in follow-up; DLE group consists of limbs with a limb embolism in follow-up.

c The OR (95% CI) and B resulting from the GEE for TI were obtained after rearranging the variable to make steps of .05 instead of 1, considering the magnitude of TI.

* p-value indicating a difference between PG and LGO or DLE groups when <0.05, which are depicted bold in the table.
